# Changes in white matter microstructure following serial ketamine infusions in treatment resistant depression

**DOI:** 10.1002/hbm.26217

**Published:** 2023-01-30

**Authors:** Brandon Taraku, Roger P. Woods, Michael Boucher, Randall Espinoza, Mayank Jog, Noor Al‐Sharif, Katherine L. Narr, Artemis Zavaliangos‐Petropulu

**Affiliations:** ^1^ Department of Neurology University of California Los Angeles Los Angeles California USA; ^2^ Department of Psychiatry and Behavioral Sciences University of California Los Angeles Los Angeles California USA

**Keywords:** antidepressant treatment, diffusion weighted, ketamine, magnetic resonance imaging, major depressive disorder, NODDI, structural connectivity

## Abstract

Ketamine produces fast‐acting antidepressant effects in treatment resistant depression (TRD). Though prior studies report ketamine‐related changes in brain activity in TRD, understanding of ketamine's effect on white matter (WM) microstructure remains limited. We thus sought to examine WM neuroplasticity and associated clinical improvements following serial ketamine infusion (SKI) in TRD. TRD patients (*N* = 57, 49.12% female, mean age: 39.9) received four intravenous ketamine infusions (0.5 mg/kg) 2–3 days apart. Diffusion‐weighted scans and clinical assessments (Hamilton Depression Rating Scale [HDRS‐17]; Snaith Hamilton Pleasure Scale [SHAPS]) were collected at baseline and 24‐h after SKI. WM measures including the neurite density index (NDI) and orientation dispersion index (ODI) from the neurite orientation dispersion and density imaging (NODDI) model, and fractional anisotropy (FA) from the diffusion tensor model were compared voxelwise pre‐ to post‐SKI after using Tract‐Based Spatial Statistics workflows to align WM tracts across subjects/time. Correlations between change in WM metrics and clinical measures were subsequently assessed. Following SKI, patients showed significant improvements in HDRS‐17 (*p*‐value = 1.8 E‐17) and SHAPS (*p*‐value = 1.97 E‐10). NDI significantly decreased in occipitotemporal WM pathways (*p* < .05, FWER/TFCE corrected). ΔSHAPS significantly correlated with ΔNDI in the left internal capsule and left superior longitudinal fasciculus (*r* = −0.614, *p*‐value = 6.24E‐09). No significant changes in ODI or FA were observed. SKI leads to significant changes in the microstructural features of neurites within occipitotemporal tracts, and changes in neurite density within tracts connecting the basal ganglia, thalamus, and cortex relate to improvements in anhedonia. NODDI may be more sensitive for detecting ketamine‐induced WM changes than DTI.

## Graphical abstract

We sought to examine ketamine's effect on white matter microstructure in patients with treatment resistant depression. By using diffusion‐weighted MRI and NODDI modeling, we found significant changes in white matter within occipital and temporal regions following serial ketamine infusions.

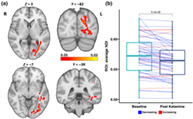

## 1 INTRODUCTION

Major depressive disorder (MDD) is a leading cause of disability worldwide and a primary contributor to the global burden of disease (*Depression*: World Health Organization, 2021). First‐line antidepressant treatments, however, do not benefit roughly one third of patients even after two or more adequately dosed trials (Gaynes et al., 2009). Ketamine, an arylcyclohexylamine derivative and *N*‐methyl‐d‐aspartate receptor (NMDAR) antagonist, is widely used as an anesthetic in medicine with a high safety profile (Bergman, 1999; Li & Vlisides, 2016). At subanesthetic dose, ketamine was more recently discovered to reduce depressive symptoms and suicidality within hours (Berman et al., 2000) with replication across multiple clinical trials (Bartoli et al., 2017; Wilkinson et al., 2018). Nonetheless, efforts to improve the sustainability of ketamine's effects, target the individuals most suited for therapy, and further drug discovery remain stifled by a relative lack of understanding concerning ketamine's antidepressant mechanism of action.

Studies focused on targeting the molecular and cellular mechanisms underlying ketamine's antidepressant effects suggest that NMDAR inhibition leads to a cascade of events that stimulate the activation of neurotrophic factors to initiate synaptic and dendritic remodeling, which subsequently modulates brain circuitry (Kokane et al., 2020). A growing body of human neuroimaging studies repeatedly demonstrate that subanesthetic ketamine treatment alters brain networks linked with mood and behavior in patients with MDD using resting state or task‐based fMRI (Alario & Niciu, 2021; Ionescu et al., 2018; Loureiro et al., 2020; Mkrtchian et al., 2021; Reed et al., 2019; Sahib, Loureiro, Vasavada, Kubicki, et al., 2020). Notably, functional brain systems are built on an architecture of direct and indirect white matter (WM) pathways (Honey et al., 2009; Horn et al., 2014) that may also contribute to ketamine's therapeutic effects. Though diffusion‐weighted imaging (DWI) is able to measure the structural integrity of neural pathways, only a few small‐sample size studies have used DWI to investigate ketamine's effect on WM microstructure in MDD. So far these DWI studies have only addressed whether WM measured pre‐treatment relates to clinical response 24 h after a single intravenous (IV) dose (Reed et al., 2019; Sydnor et al., 2020; Vasavada et al., 2016), or following serial IV ketamine (Wade et al., 2022). One study addressed whether changes in WM occur within specific bundles 4 h after a single IV dose, and relationships with clinical outcomes after 24 h (Sydnor et al., 2020). Thus, how ketamine modulates WM architecture across the whole brain over time, and how this relates to clinical outcomes remains largely unknown.

A majority of prior DWI ketamine studies have modeled WM architecture using the Diffusion Tensor (DTI) model (Basser et al., 1994). Neurite Orientation Dispersion and Density Imaging (NODDI) (Zhang et al., 2012) is a higher order diffusion model that is better suited for the analysis of multi‐shell data. Instead of modeling the diffusion tensor in three dimensions as with DTI, NODDI utilizes a multi‐compartment model capable of estimating diffusion independently in intra‐axonal space, extracellular space around axons and dendrites, and in cerebrospinal fluid (CSF). Thus, though DTI and NODDI models are both sensitive to detecting changes in WM microstructure and diffusion metrics are correlated, NODDI provides greater biophysical specificity with respect to isolating changes in neurite or axonal arrangement, dispersion, and density (Edwards et al., 2017; Fukutomi et al., 2019; Kamiya et al., 2020; Timmers et al., 2016), which has been shown to correspond with histologically derived neurite density measures (Sepehrband et al., 2015).

To understand the brain mechanisms underlying ketamine's antidepressant effects, the current study sought to determine whether neuroplasticity occurs in major WM pathways across the brain in patients with MDD following serial ketamine infusion (SKI) treatment by modeling changes in WM microstructure using NODDI. Specifically, neuroimaging and clinical assessments were collected at baseline and 24 h following four ketamine infusions (SKI) in patients with treatment resistant depression (TRD). Healthy controls were included to examine cross‐sectional differences WM microstructure, and establish if any observed ketamine‐related WM changes in patients trended toward control values. A subset of healthy controls were scanned at two timepoints over a similar time‐frame without receiving ketamine treatment to evaluate the stability of effects over time. To estimate changes in WM microstructure, neurite density and neurite orientation dispersion measures were derived from NODDI, and fractional anisotropy (FA) was derived from the DTI model. Tract‐Based Spatial Statistics (TBSS) workflows (Smith et al., 2006) aligned major WM pathways across time and subjects. Though few ketamine studies have used DWI to evaluate changes in WM microstructure in TRD (Zavaliangos‐Petropulu et al., 2022), WM structural connectivity serves to constrain functional connectivity, at least in part (Honey et al., 2010). Since prior studies generally find that ketamine increases functional connectivity between large‐scale networks such as the default mode and frontal–parietal network with nodes involved in emotion and reward processing (e.g., subgenual anterior cingulate, anterior insula, striatum, amygdala, and habenula) (Abdallah et al., 2017; Gärtner et al., 2019; Mkrtchian et al., 2021; Rivas‐Grajales et al., 2021; Rush et al., 2003; Siegel et al., 2021) in TRD, we expected to find increases or decreases in neurite/axonal dispersion (ODI) and density (NDI) in tracts connecting these regions.

## 2 METHODS

### 2.1 Subjects

Participants included 57 individuals with MDD (49.12% female, mean age, 39.9 ± 11.0 SD) who met criteria for TRD, and 49 healthy controls (HC, 53.06% female, mean age: 31.8 ± 11.5 SD). TRD was defined as an unsuccessful response to ≥2 prior antidepressant trials of sufficient dose and duration with the current episode lasting ≥6 months (Nemeroff, 2007; Sackeim, 2001). Participants included those with moderate to severe major depression as evaluated by clinical consultation using DSM‐V criteria (American Psychiatric Association, 2022). Exclusion criteria included active suicidality, comorbid substance abuse in the last 3 months, schizophrenia/schizoaffective Axis I diagnosis, psychotic disorder due to general medical condition, history of psychotic reactions to medications, history of convulsions/withdrawal seizures and having received any neuromodulation treatment or ketamine within the past 6 months. Exclusion criteria for HCs included any prior diagnosis of depression or current use of psychotropic medication and substance abuse/dependence history. Exclusion criteria for all participants included presence of neurological/physical/developmental disorders, and contraindications to scanning including pregnancy. Participants with incomplete or poor quality data were also excluded. Poor quality data was defined by scans with visible artifacts, or scans with excessive motion (average absolute motion >10 mm) determined by FSL's *eddy_qc* (Bastiani et al., 2019) (https://fsl.fmrib.ox.ac.uk/fsl/fslwiki/eddyqc/UsersGuide), and were subsequently removed. Incomplete data was defined by any TRD participant that did not complete both scan timepoints or had any volumes missing from their DWI scans. Consequently, two subjects were excluded from the TRD cohort due to excessive motion in one or more of their scans. Eight TRD subjects that did not complete either their baseline or post‐treatment scan were also removed. Additionally, two HC subjects were excluded due to excessive motion or missing volumes in their DWI scans. All participants were recruited from the Southern California area and were consented for participation as approved by the UCLA Institutional Review Board.

### 2.2 Ketamine treatment

Patients were permitted to remain on stable antidepressant medications if unchanged for ≥6 weeks prior to treatment. Benzodiazepines, which can influence cortical excitability and ketamine response (Wilkowska et al., 2021), and other medications considered a contraindication to ketamine, were discontinued during the treatment trial. Racemic ketamine was administered as 40‐min IV infusions (0.5 mg/kg) diluted in 60 cc of saline with continuous clinical and hemodynamic monitoring. Psychotomimetic effects, blood pressure, blood oxygen saturation, heart rate, and respiratory rate were monitored during and for 3 h post infusion. Patients received a total of 4 ketamine infusions, each scheduled 2 days apart. Healthy controls did not receive ketamine.

### 2.3 Study time points

**FIGURE 1:**
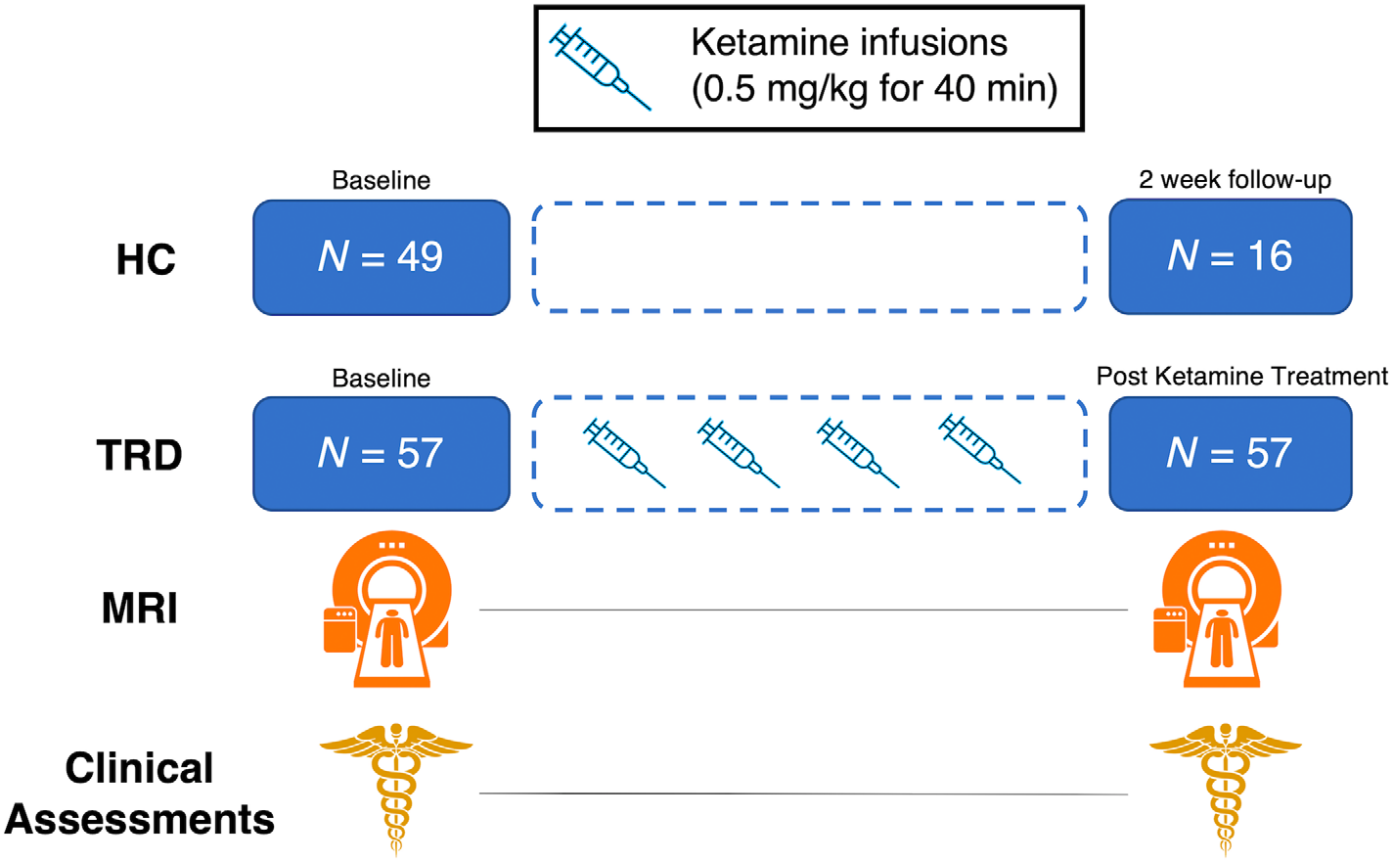
Illustration of study design. Treatment Resistant Depression (TRD) patients received clinical assessments and MRI scans at baseline and 24 h after their fourth ketamine infusion. All healthy controls (HC) were scanned once at baseline, and a subset were scanned after 2 weeks without receiving any clinical interventions

This investigation focused on examining ketamine's effects on the microstructure of WM connecting the brain's functional systems, and patients were studied using a naturalistic design without a placebo‐control. Patients were scanned and received clinical assessments at baseline (within a week of initiating treatment), and again 24 h after receiving their fourth IV infusion (Figure 1). Controls were scanned once, except for a subset of 16 participants who received a follow‐up scan approximately 2 weeks later, similar to the time interval of scanning for patients receiving SKI.

### 2.4 Clinical assessments

The Hamilton Depression Rating scale (HDRS) (Hamilton, 1960) 17‐item was used to determine relationships between DWI metrics and clinical response. Since ketamine has been shown to impact anhedonia and other motivation‐related behaviors (Ballard et al., 2017; Nogo et al., 2022) the Snaith‐Hamilton Pleasure Scale (SHAPS) (Snaith et al., 1995) was used as a secondary outcome measure. HDRS consisted of 17 questions, with total scores ranging from 0 to 52, and higher scores indicating increased depressive symptoms. SHAPS consisted of 14 questions and was scored on a 4‐point likert scale, with total scores ranging from 14 to 56 and lower scores indicating greater anhedonia.

### 2.5 MRI data acquisition

Participants were scanned using a Siemens 3 T Prisma MRI System (Erlangen, Germany) at UCLA's Ahmanson‐Lovelace Brain Mapping Center using a 32‐channel head coil. Imaging sequences were identical to those used by the Human Connectome Project (HCP) Lifespan studies for Aging and Development (Harms et al., 2018). Structural scans consisted of a T1‐weighted (T1w) multi‐echo MPRAGE (voxel size (VS) = 0.8 mm isotropic; repetition time (TR) = 2500 ms; echo time (TE) = 1.81: 1.79: 7.18 ms; inversion time (TI) = 1000 ms; flip angle (34) = 8.0°; acquisition time (TA) = 8: 22 min) and a T2‐weighted (T2w) acquisition (VS = 0.8 mm isotropic; TR = 3200 ms; TE = 564 ms; TA = 6: 35 min), both with real‐time motion correction (Tisdall et al., 2016). Diffusion MRI scans (VS = 1.5 mm isotropic; TR = 3230 ms; TE = 89.20 ms; TA = 5: 42 min) were collected using a multiband, echo‐planar imaging (EPI) sequence. Four consecutive runs of diffusion MRI were collected with reverse phase‐encoding (posterior–anterior [PA] and anterior–posterior [AP]) for each pair. Each run contained interleaved shells with two diffusion weightings (*b* = 1500 and 3000 s/mm^2^), comprising 185 diffusion directions in total across both scans within each phase encoding direction (Harms et al., 2018).

### 2.6 MRI data analysis

Imaging data was processed using the HCP minimal preprocessing pipelines (Glasser et al., 2013). Briefly, preprocessing steps implemented with FSL tools included intensity normalization of b0 volumes across runs, correcting EPI and eddy‐current induced distortions, correcting for motion and gradient non‐linearities, and registration of DWI scans to T1w space. Diffusion images were visually inspected for artifacts and head motion, and FSL's *eddy_qc* (Bastiani et al., 2019) was used to estimate average absolute motion for each image.

Following QC, the NODDI and DTI models were separately fitted to the diffusion data. The NODDI model was applied using the *Dmipy* toolbox (Fick et al., 2019) (https: //github.com/AthenaEPI/dmipy), following the multi‐tissue NODDI modeling approach (https://nbviewer.org/github/AthenaEPI/dmipy/blob/master/examples/example_multi_tissue_noddi.ipynb), which implements a response function based on the multi‐tissue constrained spherical deconvolution model (Jeurissen et al., 2014). The primary outputs of this model include the Neurite Density Index (NDI), which describes the density of axons in WM ranging from 0 (low density) to 1 (high density), and the Orientation Dispersion Index (ODI), which describes the degree of dispersion of fiber bundles due to crossing or fanning fibers, ranging from 0 (highly parallel fibers) to 1 (highly dispersed fibers). ODI and NDI are then multiplied by the WM volume fraction estimated using the multi‐tissue response, to obtain the WM signal contribution for each NODDI parameter. In an independent processing stream, the DTI model was fit to the data using FSL's DTIFit (https://fsl.fmrib.ox.ac.uk/fsl/fslwiki/FDT/UserGuide#DTIFIT), which calculates FA at every voxel.

To optimally align WM within subjects across time, TBSS was applied to the longitudinal data following a protocol outlined in a previous study (Engvig et al., 2012). In brief, both FA timepoint images per subject were co‐registered together, and a single subject template was created by averaging both co‐registered images and smoothing this image with a 1 mm kernel. The single subject templates were subsequently used to estimate nonlinear transformations to standard space for both timepoints during TBSS processing. Subjects with only one time point were directly registered to standard space. All TBSS processing steps followed the recommended protocol outlined by FSL (https://fsl.fmrib.ox.ac.uk/fsl/fslwiki/TBSS/UserGuide), which includes using FSL's *FMRIB58_FA* image as a template for standard space registration, and thresholding the WM skeleton at 0.2.

For NODDI data, the transformations to co‐register each subject's FA timepoints were applied to each subject's ODI and NDI images. Once NODDI data was co‐registered, FSL's *tbss_non_FA* was applied to bring all ODI and NDI images into standard WM skeleton space for statistical analyses.

### 2.7 Statistical analysis

To test for improvements in depressive symptoms following SKI, paired *t*‐tests were computed for each clinical assessment (HDRS, SHAPS) in TRD subjects. Differences in age and education level, or sex between TRD and HC groups were evaluated with two‐sample *t*‐tests and chi‐square tests, all performed using R version 4.1.3.

FSL's *Randomise* (Winkler et al., 2014) was used for voxel‐wise analysis of the WM skeleton for NDI, ODI, or FA, using 5000 randomly generated permutations, spatially corrected using Threshold Free Cluster Enhancement (TFCE) (Smith & Nichols, 2009), and family‐wise error rate (FWER) correction. All analyses in *Randomise* included age and sex as covariates of no interest. To test for the longitudinal effects of SKI on WM microstructure, a “difference” image was computed for each TRD subject (baseline image subtracted from post‐treatment image), and was used as input for a voxel‐wise 1‐sample *t*‐test in *Randomise*.

To investigate if pre‐to‐post treatment changes in WM were associated with changes in clinical assessments, a voxel‐wise correlation analysis was performed in *Randomise*, which calculated the correlation between percent change in WM (difference image divided by baseline image for each subject) and percent change in each clinical assessment (HDRS, SHAPS). Voxels which were significant at *p* < .05 after FWER and TFCE correction were determined to be significantly correlated with improvements in mood.

For visualization purposes, significantly correlated voxels (FWER and TFCE corrected) were used to create a region of interest (ROI), and the Johns Hopkins University (JHU) ICBM atlas (Hua et al., 2008) was used to further parcellate the ROI into specific WM tracts of interest, such that percent change within each section of the WM tract could be calculated and graphed to visually show associations with changes in mood. Finally, in a supplementary analysis, the entire WM skeleton was parcellated into 48 tract ROIs from the JHU atlas to examine associations between percent change at every WM tract ROI and clinical score, correcting for multiple comparisons (Bonferroni: 0.05/48 = *p* < .00104). All correlations were calculated using partial correlations controlling for age and sex, implemented in MATLAB R2020b.

For cross‐sectional analysis of diffusion measures between TRD and HC groups, a two‐sample *t*‐test was implemented in *Randomise* using baseline images from each TRD and HC participant as input.

In a supplementary analysis, an ANOVA was performed where changes in the longitudinal HC subsample were compared to changes in TRD patients following SKI both within WM tracts shown to change significantly in patients, and at the voxel level. Results of these analyses are shown in Figure S3.

## 3 RESULTS

### 3.1 Demographic and clinical results

**FIGURE 2:**
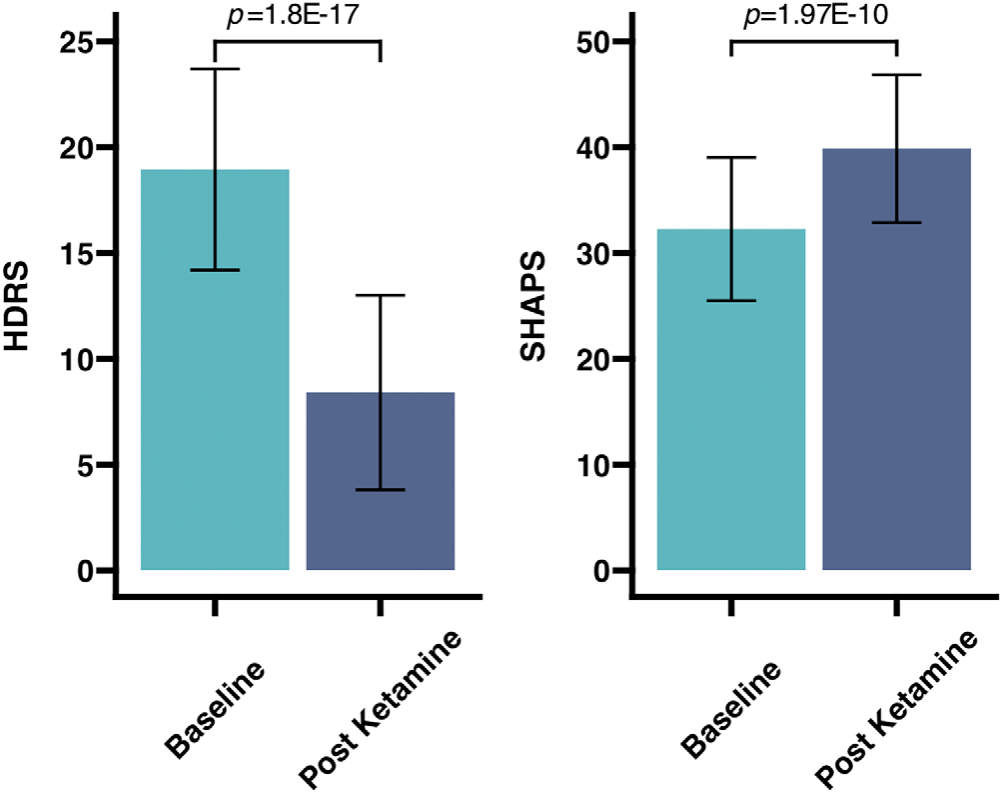
Changes in clinical measures following Serial Ketamine Infusion. TRD patients showed significant improvements in overall depressed mood measured using HDRS, and anhedonia measured using SHAPS, following treatment. HDRS scores can range from 0 to 52 with higher scores indicating more severe depression, whereas SHAPS scores can range from 14 to 56 with lower scores indicating more severe anhedonia. Bar graphs show the mean scores and confidence intervals in the TRD group at baseline and post treatment, with *p*‐values from paired *t*‐tests labeled above. HDRS, Hamilton Depression Rating Scale; SHAPS, Snaith Hamilton Pleasure Scale; TRD, treatment resistant depression

The demographic and clinical characteristics of all participants are summarized in Table 1. Patients with TRD showed significant improvements in both HDRS (*t* = −12.25, *p* = 1.8E‐17), and SHAPS (*t* = 7.75, *p* = 1.97E‐10) scores following SKI (Figure 2). Of the 57 patients that completed SKI, 29 reached remission status (HDRS ≤ 7). Sex (χ^2^ = 1.18, *p* = .277) and education level (*t* = −0.58, *p* = .57) did not significantly differ between the TRD and HC group. However, healthy controls were significantly younger than TRD patients (*t* = 3.7, *p* = .00035).

**TABLE 1:** Demographic and clinical information

	HC mean (SD)	MDD mean (SD)	*t*/χ^2^	*p*
Number of subjects (*N*)	49	57	–	–
Sex (% female)	53.06	49.12	χ^2^ = 1.137	.286
Age (years)	31.8 (11.5)	39.89 (11.0)	*t* = 3.00	.0035
Education (ISCED variable)	6.08 (1.04)	5.89 (1.19)	*t* = −0.58	.57
Duration of lifetime illness (years)	–	23.50 (12.41)	–	–
Current episode (years)	–	4.42 (6.11)	–	–
Race (% Asian)	20.4	10.5	–	–
Race (% Black)	20.4	0	–	–
Race (% Hawaiian or Pacific Islander)	2	0	–	–
Race (% more than one race)	4.1	1.8	–	–
Race (% unknown/not reported)	10.2	7	–	–
Race (% White)	42.9	80.7	–	–

### 3.2 Longitudinal effects of ketamine on white matter structure

**FIGURE 3:**
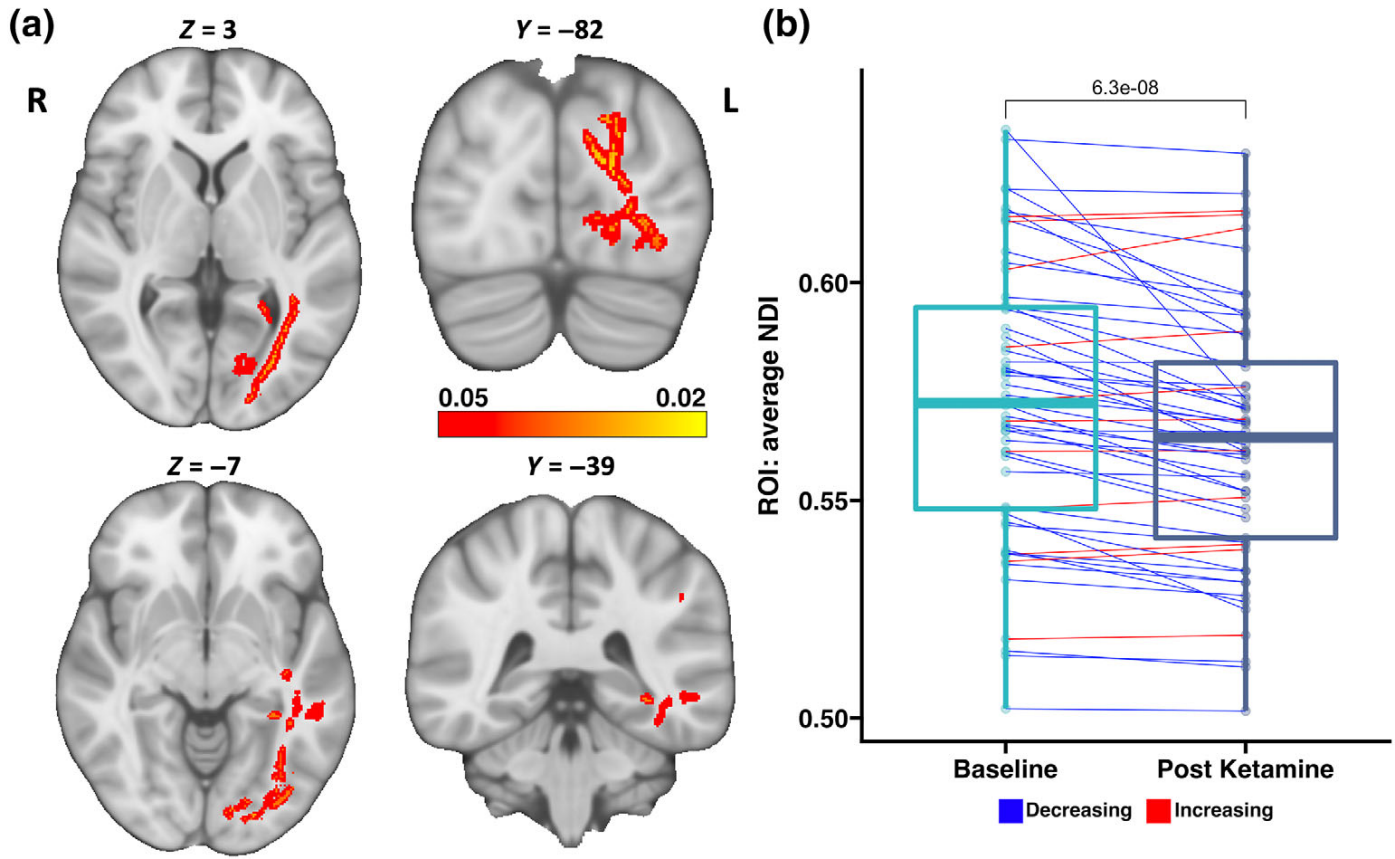
Significant changes in WM following Serial Ketamine Infusion. Significant decreases in the NDI were observed in tracts connecting temporal, occipital and limbic regions, including the left posterior thalamic radiation, left inferior longitudinal fasciculus, and left retrolenticular region of the internal capsule. (a) Multi‐slice views of WM regions where NDI was significantly reduced, overlaid on an MNI‐152 T1w brain with MNI coordinates for each slice view. Brighter colors indicate greater significance. (b) Using the significant WM tracts as a statstical ROI, the average NDI values were calculated within this ROI before and after ketamine treatment for each subject. Boxplots for each timepoint show the distribution of average NDI values, and lines connecting subjects at both timepoints show change in average NDI for each subject following ketamine treatment (blue = decrease, red = increase). A *p*‐value (6.3E‐08) from a paired *t*‐test comapring average NDI change within the ROI over time is listed above the boxplots. NDI, Neurite Density Index; ROI, region of interest; WM, white matter

Significant decreases in NDI (*p* < .05, FWER and TFCE corrected) in major WM tracts within the left occipital and left temporal lobes, including the posterior thalamic radiation, inferior longitudinal fasciculus, forceps major, and the retrolenticular part of the internal capsule were observed pre‐to‐post SKI (Figure 3). No significant changes were found for ODI or FA following SKI in TRD.

No significant changes in any of the diffusion metrics were observed in the subset of HC subjects with longitudinal scans.

### 3.3 Relationships with clinical outcome

**FIGURE 4:**
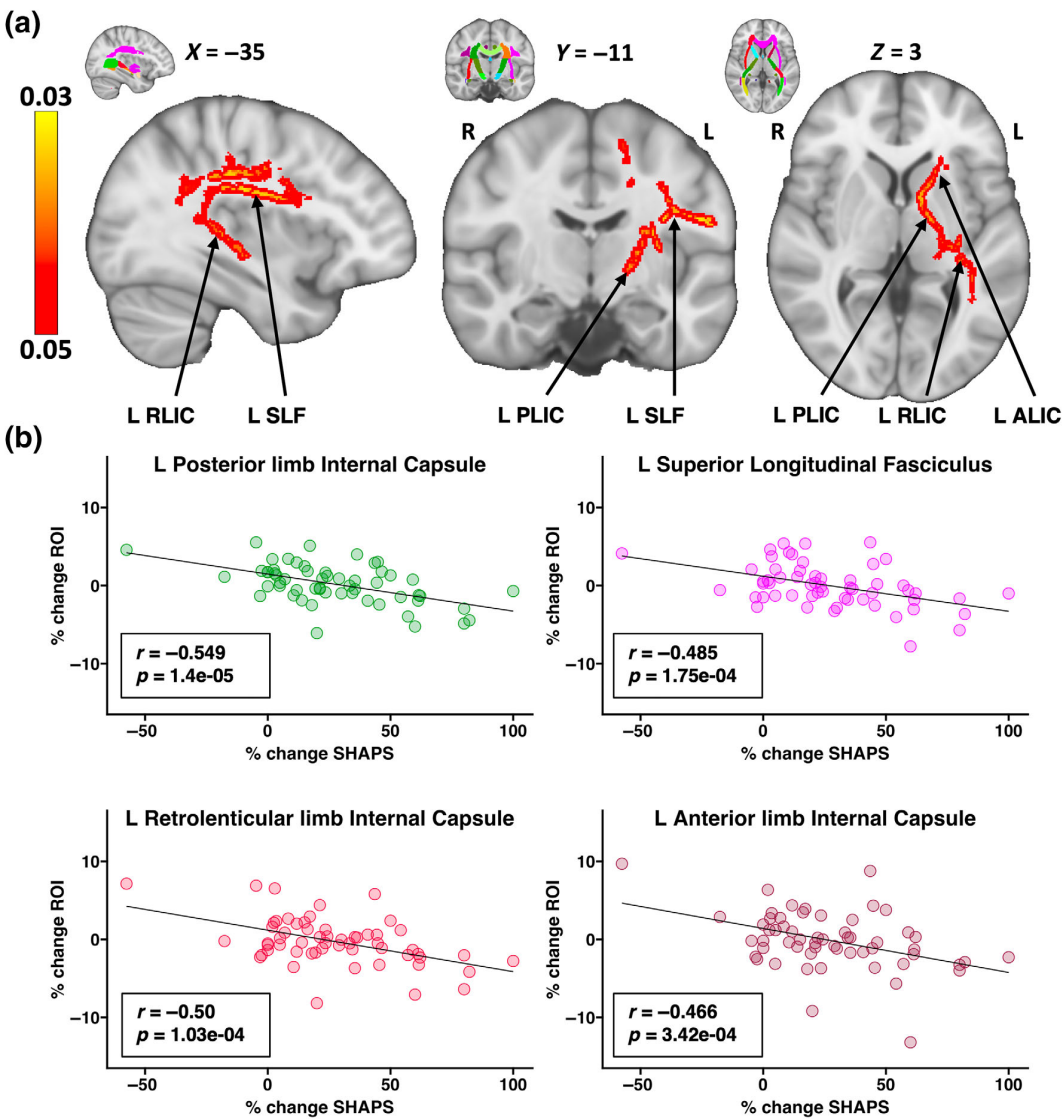
WM Associations with SHAPS following Serial Ketamine Infusion. Percent change in SHAPS was significantly negatively correlated with percent change in NDI within tracts passing through the left superior longitudinal fasciculus and left internal capsule, suggesting that greater NDI reductions correspond to greater improvements in anhedonia. The average percent NDI change within the entire statistical ROI (*p* < .05 TFCE and FWER corrected) showed a significant correlation with percent change in SHAPS (*r* = −0.614, *p* = 6.24E‐09). The JHU atlas was then used to parcellate the statistical ROI into specific tracts of interest, in order to visualize how NDI change within each tract segment contributes to this observed relationship. (a) Slice views of MNI‐152 T1w brain image with significantly correlated voxels overlaid on top. MNI slice coordinates are displayed above each slice. The top right corner of each slice shows an identical slice with the JHU atlas overlaid for reference of where ROIs are defined. (b) Scatter plots of the four tract ROIs from parcellating the statistical ROI, which includes three segments of the left internal capsule, and the left superior longitudinal fasciculus. Since there is one extreme value in the scatter plots, we repeated the correlation analysis after removing this outlier to ensure that the effects are not entirely driven by this one data point. Results remain significant at the voxel level after FWER and TCFE correction as shown in Figure S4. *R* and *p* values are listed on each plot. Scatter plots are color‐coded to match the color on the JHU atlas. ALIC, anterior limb of the internal capsule; FWER, family‐wise error rate; JHU, Johns Hopkins University; L, left; NDI, Neurite Density Index; PLIC, posterior limb of the internal capsule; R, right; RLIC, retrolenticular limb of the internal capsule; ROI, region of interest; SHAPS, Snaith Hamilton Pleasure Scale; SLF, superior longitudinal fasciculus; TFCE, Threshold Free Cluster Enhancement

At the voxel level, greater decreases in NDI in WM tracts passing through the left internal capsule and left superior longitudinal fasciculus significantly correlated with greater improvements in SHAPS (*p* < .05, FWER and TFCE corrected) (Figure 4).

Correlations between change in diffusion derived measures averaged across all WM tracts from the JHU atlas (independent of the statistical maps) and change in clinical assessments are provided in Table S1.

### 3.4 Cross‐sectional effects of diagnosis on white matter structure

No significant differences in NDI were observed between TRD and HC groups. However, significant differences in ODI and FA (*p* < .05 FWER and TFCE corrected) were detected where patients with TRD exhibited higher ODI and lower FA in broadly distributed and overlapping WM tracts, including bilateral brainstem, and cerebellar tracts, corpus callosum, cingulum, inferior longitudinal fasciculus, posterior thalamic radiation and uncinate fasciculus, and the left superior longitudinal fasciculus, external capsule, and internal capsule.

## 4 DISCUSSION

This investigation sought to determine whether serial subanesthetic IV ketamine therapy leads to neuroplasticity in major WM pathways in patients with TRD, and whether observed changes in WM microstructure are associated with improvements in mood and anhedonia. To accomplish this goal, we used a multi‐shell DWI sequence with high spatial resolution (1.5 mm isotropic) developed by the HCP (Harms et al., 2018), and implemented the NODDI model to estimate neurite density and dispersion in each voxel within WM tracts. Comparing voxel‐wise changes in neurite density, we observed significant changes in WM microstructure within left hemisphere occipitotemporal tracts including the inferior longitudinal fasciculus, forceps major, posterior thalamic radiation, and the retrolenticular part of the internal capsule, 24 h after patients had completed the fourth ketamine infusion. Additionally, we observed significant associations between decreases in neurite density and improvements in anhedonia within the left superior longitudinal fasciculus and left internal capsule. We did not find significant differences in voxel‐wise comparisons of NDI between TRD patients and HC participants, however, we observed significant cross‐sectional findings for ODI and FA between diagnostic groups.

### 4.1 Ketamine‐related modulation of white matter structure

Regarding longitudinal effects of SKI on WM microstructure, we found significant decreases in NDI in left hemisphere tracts including the posterior thalamic radiation, inferior longitudinal fasciculus, forceps major and retrolenticular part of the internal capsule. Altered microstructural properties of WM pathways serving to connect functional brain networks are consistently reported in patients with depression (Coloigner et al., 2019; Drevets et al., 2008; Liao et al., 2013; Murphy & Frodl, 2011; Van Velzen et al., 2020). Previous studies have also shown that changes in WM microstructure relate to, or may serve as biomarkers of therapeutic response to other antidepressant treatments (Bracht et al., 2015; Davis et al., 2019; Lyden et al., 2014), which may indicate how a particular intervention engages brain circuitry to guide more effective personalized treatment strategies. Two preliminary studies have specifically addressed how variations in pretreatment FA relate to clinical response following single ketamine treatment (Sydnor et al., 2020; Vasavada et al., 2016). Both studies suggest that greater FA in the cingulum (Sydnor et al., 2020; Vasavada et al., 2016), superior longitudinal fasciculus (Sydnor et al., 2020) or forceps major (Vasavada et al., 2016) present a biomarker for improved clinical outcomes following single dose ketamine. However, clinical (Sydnor et al., 2020) and preclinical (Geiger et al., 2021) studies have so far either only examined or reported changes in diffusion properties during and immediately following ketamine administration. To the best of our knowledge, this study is the first to investigate changes in WM microstructure following serial ketamine treatment, and to utilize NODDI in this context. These changes suggest the occurrence of neuroplasticity following ketamine treatment.

Amongst the few prior DWI ketamine studies published, majority have used the diffusion tensor model, which remains limited with regard to the complex arrangement of dendrites and axons and crossing fibers (Jeurissen et al., 2013). Consequently, these measures may lack microstructural specificity, since FA can be influenced by both the orientation dispersion and density of neurites (Zhang et al., 2012). Our findings revealed significant decreases in neurite density 24 h after SKI, while changes in FA were below the threshold of significance (though trended in the same direction), which suggest that neurite density may provide a more sensitive treatment‐related biomarker of ketamine.

Our observations of decreases in neurite density following ketamine, primarily in the occipital and temporal lobes, may suggest a decrease in structural connectivity between regions and suggest some consistency with previous SKI fMRI research. For example, in a sample overlapping with this study, Sahib, Loureiro, Vasavada, Kubicki, et al. (2020) reported decreases in the BOLD signal in left visual, superior parietal, and temporal regions, which all receive connections from the WM tracts implicated in the current investigation. Furthermore, another related fMRI study investigated the effect of SKI on resting‐state functional connectivity across the whole brain, and found that MDD participants exhibited higher functional connectivity than controls between V1 and a node of the ventral attention network within the temporal lobe, which decreased towards healthy controls following ketamine treatment (Sahib, Loureiro, Vasavada, Anderson, et al., 2020). Of relevance, prior MDD studies have reported increased motion perception and consequently decreased spatial suppression on a visual perception task (Golomb et al., 2009) as well as deficits in visual input integration in MDD (Zomet et al., 2008), which may be influenced by GABA‐ergic neurotransmission in the visual cortex (Du et al., 2022; Song et al., 2021). Thus, ketamine‐related changes in WM tracts connecting temporal and occipital regions may have a yet to be specified neuropsychological correlate.

In this study we found reductions in NDI after SKI. Decreases in NDI are often associated with signs of neurodegeneration, such as in Alzheimer's disease progression (Raghavan et al., 2021) and traumatic brain injury (Palacios et al., 2020). Simultaneously, in healthy populations, decreased neurite density may reflect processes involved in neural reorganization that improve the connectivity of functional systems, such as through neurite pruning (Kondo et al., 2017). Since we cannot infer the precise biological meaning underlying the observed changes in neurite density, future interdisciplinary studies may be necessary to clarify the underlying functional relevance of the observed NDI changes following ketamine. Irrespective, our results suggest that ketamine treatment perturbs networks involved in higher‐level visual processing and potentially other depression‐relevant networks in TRD.

### 4.2 Associations with clinical response

Since longitudinal effects may indicate biological processes associated with ketamine treatment independent of its antidepressant effects, we also investigated if any WM changes were associated with overall clinical response (HDRS), and anhedonia (SHAPS) specifically. Although MDD diagnosis can include a large variation in clinical symptoms (De Fruyt et al., 2020), anhedonia, or loss of interest or pleasure, must be present for diagnosis. Anhedonia appears notoriously difficult to treat, often remaining when other depression symptoms are reduced following conventional treatments (Cao et al., 2019; Treadway & Zald, 2011). In contrast, ketamine is shown to significantly improve anhedonic symptoms (Nogo et al., 2022; Rodrigues et al., 2020), even independently of overall mood (Lally et al., 2014).

Anhedonia is thought to reflect disturbances in reward processing circuitry involving prefrontal, striatal and limbic structures (Höflich et al., 2019; Keedwell et al., 2005). In this study, we found that decreased NDI in WM tracts, primarily the left internal capsule and left superior longitudinal fasciculus, associated with improvements in anhedonia. Notably, the internal capsule, previously used as a target for deep brain stimulation in TRD (Widge et al., 2019), forms an integral part of the frontostriatal network involved in reward processing. Furthermore, disturbances in this network are repeatedly reported in MDD and have been linked to symptoms of anhedonia (Furman et al., 2011). Several studies have also specifically reported changes in frontostriatal connectivity following ketamine treatment (Gärtner et al., 2019; Mkrtchian et al., 2021; Siegel et al., 2021), with one study reporting correlations between increased frontostriatal resting state connectivity and improvements in anhedonia (Mkrtchian et al., 2021). Though in an fMRI study investigating adolescent patients with TRD, greater reductions in corticolimbic and corticostriatal network activation during an emotional task were associated with decreases in anhedonia following single ketamine (Thai et al., 2020). Yet another study found hyperconnectivity between striatal and limbic regions with the default mode network correlated with increased anhedonia (Hwang et al., 2016). These somewhat conflicting findings from the functional imaging literature underscore the need to understand the relationships between changes in structural and functional brain circuitry or connectomes. Regardless, the current findings further suggest that ketamine modulates limbic and reward circuitry, and indicate that microstructure of WM pathways within these networks contribute to ketamine's anti‐anhedonic properties.

In the current investigation, we also found that changes in the left superior longitudinal fasciculus (SLF) are associated with improvements in anhedonia. The SLF connects frontal, temporal, parietal and occipital cortices. Previous studies have shown reduced SLF FA in MDD patients relative to healthy controls (Jiang et al., 2017; Lai & Wu, 2014), which could potentially contribute to psychomotor retardation, language processing or memory deficits frequently associated with MDD (Jiang et al., 2017). Using an overlapping sample with our current work, Wade et al., 2022 (Wade et al., 2022) used a data‐driven approach to investigate whether multimodal MRI measures at baseline predict treatment response to ketamine in MDD. Results showed that decreased diffusion kurtosis (DK) in the SLF was predictive of greater improvement in anhedonia following SKI. Interestingly, an independent study also found that greater FA in the SLF was associated with increased anhedonia (Coloigner et al., 2019). These observations show some overlap with our findings, and together support that changes in microstructural features in the SLF may contribute to the improvements in anhedonic symptoms following SKI.

### 4.3 Cross‐sectional differences of white matter structure

While we failed to observe significant differences in NDI between our TRD group and controls, we found significant differences in ODI and FA between diagnostic groups. TRD patients were found to have higher ODI and lower FA than controls in bilateral brainstem and cerebellar tracts, corpus callosum, cingulum, inferior longitudinal fasciculus, posterior thalamic radiation, and uncinate fasciculus, and left superior longitudinal fasciculus, external capsule and internal capsule. This finding partially overlaps with prior NODDI research that found MDD participants exhibited higher ODI in bilateral superior longitudinal fasciculus and left posterior thalamic radiation when compared to healthy controls (Ota et al., 2018) The same study also found that MDD participants showed lower NDI in the left middle cerebellar peduncle (Ota et al., 2018), a finding we could not replicate in our sample. Given that this current study was not explicitly powered to address cross‐sectional effects (Van Velzen et al., 2020) and has a significant age difference between groups, cross‐sectional findings should be considered preliminary.

## 5 LIMITATIONS

The current study includes naturalistic design without a placebo group. Though the objective here was to investigate neurobiological effects of SKI rather than its efficacy, the inclusion of a clinical control group receiving placebo or other antidepressant treatment is required to clearly ascribe the neurobiological changes observed to SKI. Notably, there was also a significant interaction in WM regions showing significant change in patients post‐ketamine compared to HCs scanned twice, although these results did not survive FWER correction when compared at the voxel level (Figure S3). Additionally, patients were permitted to remain on concomitant antidepressant treatment, which despite remaining stable and using a within‐subjects design, may have interacted with ketamine to influence treatment outcomes. Since we did not find significant cross‐sectional differences in NDI between our TRD and HC groups, we also cannot infer whether observed changes following ketamine normalize towards patterns typical of healthy controls. However, we note that this study was not designed for cross‐sectional analyses, which may require very large sample sizes (Marek et al., 2022; Van Velzen et al., 2020), and instead designed to investigate the longitudinal effects of ketamine. Furthermore, future follow up studies may be needed to decipher the biological relevance underlying the observed changes in neurite density. Finally, few NDI‐specific studies exist with which to compare our results. However, our findings suggest NODDI may be more sensitive than DTI for detecting microstructural changes associated with ketamine, adding support for the use of NODDI in future MDD treatment studies.

## 6 CONCLUSION

In this study, we show that ketamine modulates WM microstructure within left visual, temporal and limbic regions, using NODDI to quantify WM changes. Overall, these findings suggest microstructural changes within WM tracts connecting specific functional networks may contribute to the therapeutic effects of ketamine, and to improvements in anhedonia specifically.

## CONFLICTS OF INTEREST

The authors declare they have no disclosures or conflicts of interest.

## Supporting information


**Table S1:** Associations with clinical scores and JHU atlas ROIs
**Figure S1:** Additional slice views of the significant changes in WM following serial ketamine infusion (SKI), which expands upon the images shown in Figure 3a. Significant voxels are overlaid on the MNI‐152 brain, with brighter colors corresponding to greater significance.
**Figure S2:** Additional slice views of the regions that showed significant associations with SHAPS following serial ketamine infusion, which expands upon the images shown in Figure 4a. Significant voxels are overlaid on the MNI‐152 brain, with brighter colors corresponding to greater significance.
**Figure S3:** Comparisons between WM NDI changes in healthy controls over time versus NDI changes in treatment resistant depression following serial ketamine treatment. (a) A voxel‐level two‐sample *t* test was performed in *Randomise* to compare NDI change in TRD and HC participants, using age and sex as covariates of no interest. Though voxel‐level results of change between HCs and TRD did not survive FWER correction, uncorrected p‐value maps with TFCE showed trends in WM regions overlapping with those showing significant decreases following ketamine in TRD (Figure 3). (b) Using voxels showing significant effects of ketamine as a statistical ROI, NDI change was computed for each subject and compared between the TRD and HC groups. A significant difference between groups was observed within this statistical ROI (*t* = −2.954, *p* = .0043), with TRD patients showing significantly greater decreases in NDI than HC participants. Boxplots show NDI for TRD and HCs at each timepoint, with lines connecting each subject at both timepoints to indicate the change over time. *p*‐Values comparing change across time within each group are shown above each set of box plots.
**Figure S4:** WM Associations with SHAPS following serial ketamine infusion after removal of outliers. The voxel‐wise correlation analysis was repeated after the removal of a single outlier participant. Change in NDI within the left superior longitudinal fasciculus remains significantly negatively correlated with SHAPS, suggesting that greater reductions in NDI within these tracts are associated with greater improvements in anhedonia following ketamine treatment. (a) Slice views of MNI‐152 T1w brain image with significantly correlated voxels overlaid on top, and coordinates for each slice below. (b) The average percent change in NDI within the significant voxels was computed for each subject and plotted against their percent change in SHAPS in order to visualize the associations between change in NDI and anhedonia. *R*‐ and *p*‐values are shown above for the correlation between percent change in NDI averaged across significant voxels and percent change in SHAPS.

## Data Availability

The data that support the findings of this study are expected to be publicly available in June 2024 in the NIMH Data Archive at https://nda.nih.gov/, in collection C2844.
