# Supplementary material for: Changes in white matter microstructure following serial ketamine infusions in treatment resistant depression
Source: Hum Brain Mapp. 2023 Jan 30;44(6):2395–406. doi: 10.1002/hbm.26217 (PMC10028677; doi:10.1002/hbm.26217)
Supplement: Supplementary file 1 — Table S1: Associations with clinical scores and JHU atlas ROIs Figure S1: Additional slice views of the significant changes in WM following serial ketamine infusion (SKI), which expands upon the images shown in Figure 3a. Significant voxels are overlaid on the MNI‐152 brain, with brighter colors corresponding to greater significance. Figure S2: Additional slice views of the regions that showed significant associations with SHAPS following serial ketamine infusion, which expands upon the images shown in Figure 4a. Significant voxels are overlaid on the MNI‐152 brain, with brighter colors corresponding to greater significance. Figure S3: Comparisons between WM NDI changes in healthy controls over time versus NDI changes in treatment resistant depression following serial ketamine treatment. (a) A voxel‐level two‐sample t test was performed in Randomise to compare NDI change in TRD and HC participants, using age and sex as covariates of no interest. Though voxel‐level results of change between HCs and TRD did not survive FWER correction, uncorrected p‐value maps with TFCE showed trends in WM regions overlapping with those showing significant decreases following ketamine in TRD (Figure 3). (b) Using voxels showing significant effects of ketamine as a statistical ROI, NDI change was computed for each subject and compared between the TRD and HC groups. A significant difference between groups was observed within this statistical ROI (t = −2.954, p = .0043), with TRD patients showing significantly greater decreases in NDI than HC participants. Boxplots show NDI for TRD and HCs at each timepoint, with lines connecting each subject at both timepoints to indicate the change over time. p‐Values comparing change across time within each group are shown above each set of box plots. Figure S4: WM Associations with SHAPS following serial ketamine infusion after removal of outliers. The voxel‐wise correlation analysis was repeated after the removal of a single outlier participant. Cha [file HBM-44-2395-s001.docx]

**Supplementary Table 1: Associations with clinical scores and JHU atlas ROIs**

| **JHU tract label** | **NDI** | | | | **ODI** | | | | **FA** | | | |
| --- | --- | --- | --- | --- | --- | --- | --- | --- | --- | --- | --- | --- |
|  | **HDRS** | | **SHAPS** | | **HDRS** | | **SHAPS** | | **HDRS** | | **SHAPS** | |
|  | r | p | r | p | r | p | r | p | r | p | r | p |
| Middle cerebellar peduncle | 0.101 | 0.463 | -0.036 | 0.794 | -0.230 | 0.091 | 0.040 | 0.771 | 0.300 | 0.026 | -0.105 | 0.445 |
| Pontine crossing tract | -0.043 | 0.753 | -0.012 | 0.929 | 0.030 | 0.828 | 0.006 | 0.964 | 0.068 | 0.621 | -0.032 | 0.814 |
| Genu of corpus callosum | 0.092 | 0.506 | -0.180 | 0.188 | -0.234 | 0.086 | 0.153 | 0.264 | 0.227 | 0.096 | -0.280 | 0.038 |
| Body of corpus callosum | -0.052 | 0.706 | 0.125 | 0.365 | -0.270 | 0.046 | 0.100 | 0.469 | 0.086 | 0.534 | -0.156 | 0.256 |
| Splenium of corpus callosum | 0.147 | 0.284 | -0.213 | 0.119 | -0.195 | 0.154 | 0.026 | 0.852 | 0.302 | 0.025 | -0.225 | 0.099 |
| Fornix | 0.071 | 0.607 | -0.250 | 0.065 | -0.101 | 0.465 | -0.076 | 0.581 | 0.161 | 0.239 | -0.111 | 0.419 |
| Corticospinal tract R | -0.008 | 0.954 | -0.003 | 0.982 | -0.181 | 0.187 | -0.074 | 0.589 | 0.227 | 0.095 | 0.070 | 0.609 |
| Corticospinal tract L | -0.059 | 0.666 | -0.049 | 0.721 | -0.042 | 0.760 | 0.046 | 0.741 | 0.084 | 0.543 | -0.101 | 0.463 |
| Medial lemniscus R | 0.068 | 0.624 | 0.011 | 0.937 | -0.210 | 0.124 | -0.112 | 0.414 | 0.231 | 0.089 | 0.073 | 0.596 |
| Medial lemniscus L | 0.140 | 0.309 | -0.018 | 0.893 | -0.082 | 0.553 | 0.086 | 0.534 | 0.218 | 0.110 | -0.074 | 0.594 |
| Inferior cerebellar peduncle R | 0.058 | 0.673 | 0.098 | 0.478 | -0.261 | 0.054 | 0.215 | 0.116 | 0.246 | 0.070 | -0.173 | 0.208 |
| Inferior cerebellar peduncle L | -0.064 | 0.643 | 0.080 | 0.563 | -0.142 | 0.299 | 0.050 | 0.716 | 0.134 | 0.331 | -0.011 | 0.935 |
| Superior cerebellar peduncle R | -0.044 | 0.750 | 0.242 | 0.075 | -0.385 | 0.004 | 0.279 | 0.039 | 0.329 | 0.014 | -0.145 | 0.292 |
| Superior cerebellar peduncle L | -0.095 | 0.491 | 0.186 | 0.173 | -0.361 | 0.007 | 0.119 | 0.388 | 0.272 | 0.044 | -0.037 | 0.788 |
| Cerebral peduncle R | -0.150 | 0.275 | 0.203 | 0.138 | -0.157 | 0.252 | 0.031 | 0.825 | 0.014 | 0.920 | 0.066 | 0.632 |
| Cerebral peduncle L | -0.010 | 0.943 | -0.026 | 0.851 | -0.041 | 0.765 | 0.057 | 0.681 | -0.012 | 0.929 | -0.027 | 0.844 |
| Anterior limb of internal capsule R | 0.070 | 0.613 | -0.204 | 0.136 | -0.198 | 0.148 | 0.066 | 0.631 | 0.178 | 0.194 | -0.173 | 0.206 |
| Anterior limb of internal capsule L | 0.182 | 0.184 | -0.325 | 0.016 | -0.205 | 0.134 | 0.052 | 0.709 | 0.282 | 0.037 | -0.262 | 0.053 |
| Posterior limb of internal capsule R | 0.142 | 0.302 | -0.234 | 0.086 | -0.040 | 0.769 | -0.157 | 0.254 | 0.216 | 0.113 | -0.040 | 0.772 |
| Posterior limb of internal capsule L | 0.256 | 0.060 | **-0.430** | **0.001** | -0.029 | 0.831 | -0.092 | 0.506 | 0.266 | 0.050 | -0.319 | 0.018 |
| Retrolenticular part of internal capsule R | 0.329 | 0.014 | -0.127 | 0.356 | -0.077 | 0.574 | -0.011 | 0.936 | 0.237 | 0.081 | -0.007 | 0.959 |
| Retrolenticular part of internal capsule L | 0.209 | 0.125 | -0.314 | 0.020 | -0.079 | 0.568 | 0.028 | 0.839 | 0.281 | 0.038 | -0.309 | 0.022 |
| Anterior corona radiata R | -0.047 | 0.736 | -0.191 | 0.162 | -0.105 | 0.446 | 0.028 | 0.842 | 0.074 | 0.589 | -0.104 | 0.450 |
| Anterior corona radiata L | 0.181 | 0.186 | -0.216 | 0.114 | -0.079 | 0.568 | 0.100 | 0.468 | 0.140 | 0.310 | -0.170 | 0.213 |
| Superior corona radiata R | 0.134 | 0.328 | -0.156 | 0.256 | -0.072 | 0.602 | 0.061 | 0.661 | 0.097 | 0.482 | -0.099 | 0.473 |
| Superior corona radiata L | 0.219 | 0.108 | -0.323 | 0.016 | -0.142 | 0.302 | 0.102 | 0.458 | 0.133 | 0.331 | -0.213 | 0.118 |
| Posterior corona radiata R | 0.093 | 0.500 | -0.129 | 0.349 | -0.247 | 0.070 | 0.043 | 0.758 | 0.283 | 0.036 | -0.180 | 0.189 |
| Posterior corona radiata L | 0.075 | 0.586 | -0.059 | 0.670 | -0.261 | 0.054 | 0.243 | 0.074 | 0.234 | 0.086 | -0.197 | 0.149 |
| Posterior thalamic radiation R | 0.135 | 0.326 | -0.007 | 0.957 | -0.158 | 0.251 | 0.095 | 0.490 | 0.295 | 0.029 | -0.088 | 0.523 |
| Posterior thalamic radiation L | 0.082 | 0.552 | -0.125 | 0.363 | -0.167 | 0.223 | 0.089 | 0.516 | 0.259 | 0.056 | -0.207 | 0.129 |
| inferior longitudinal fasciculus R | -0.005 | 0.972 | 0.005 | 0.970 | -0.122 | 0.377 | -0.128 | 0.354 | 0.094 | 0.494 | 0.138 | 0.314 |
| inferior longitudinal fasciculus L | 0.121 | 0.379 | -0.062 | 0.655 | 0.057 | 0.680 | 0.067 | 0.625 | 0.116 | 0.400 | -0.088 | 0.524 |
| External capsule R | 0.111 | 0.421 | -0.156 | 0.254 | -0.218 | 0.110 | 0.025 | 0.853 | 0.207 | 0.130 | -0.041 | 0.766 |
| External capsule L | 0.186 | 0.174 | -0.261 | 0.054 | -0.073 | 0.597 | 0.098 | 0.477 | 0.187 | 0.173 | -0.242 | 0.075 |
| Cingulum (cingulate gyrus) R | 0.205 | 0.132 | 0.009 | 0.948 | -0.139 | 0.312 | 0.097 | 0.482 | 0.173 | 0.206 | -0.141 | 0.304 |
| Cingulum (cingulate gyrus) L | 0.162 | 0.238 | -0.151 | 0.272 | -0.062 | 0.654 | 0.011 | 0.936 | 0.049 | 0.723 | -0.048 | 0.727 |
| Cingulum (hippocampus) R | 0.103 | 0.455 | -0.040 | 0.771 | -0.129 | 0.346 | 0.090 | 0.516 | 0.166 | 0.225 | 0.000 | 0.998 |
| Cingulum (hippocampus) L | 0.247 | 0.069 | -0.087 | 0.526 | 0.010 | 0.943 | 0.007 | 0.961 | 0.250 | 0.066 | -0.079 | 0.569 |
| Fornix (cres) / Stria terminalis R | -0.013 | 0.923 | -0.186 | 0.173 | -0.166 | 0.226 | 0.139 | 0.313 | 0.074 | 0.593 | -0.121 | 0.378 |
| Fornix (cres) / Stria terminalis L | 0.159 | 0.246 | -0.096 | 0.484 | -0.061 | 0.658 | 0.204 | 0.135 | 0.179 | 0.191 | -0.314 | 0.020 |
| Superior longitudinal fasciculus R | 0.199 | 0.144 | -0.294 | 0.030 | -0.187 | 0.171 | 0.045 | 0.743 | 0.254 | 0.062 | -0.145 | 0.292 |
| Superior longitudinal fasciculus L | 0.285 | 0.035 | -0.351 | 0.009 | -0.129 | 0.347 | 0.018 | 0.894 | 0.299 | 0.027 | -0.273 | 0.044 |
| Superior fronto-occipital fasciculus R | 0.097 | 0.481 | -0.035 | 0.798 | -0.065 | 0.637 | 0.023 | 0.870 | 0.091 | 0.508 | -0.103 | 0.455 |
| Superior fronto-occipital fasciculus L | 0.262 | 0.054 | 0.004 | 0.980 | -0.027 | 0.846 | 0.018 | 0.894 | 0.097 | 0.481 | -0.033 | 0.808 |
| Uncinate fasciculus R | 0.001 | 0.997 | 0.071 | 0.607 | 0.005 | 0.970 | 0.036 | 0.793 | 0.043 | 0.757 | 0.008 | 0.955 |
| Uncinate fasciculus L | 0.075 | 0.588 | 0.038 | 0.782 | -0.058 | 0.676 | -0.138 | 0.315 | 0.058 | 0.672 | 0.097 | 0.481 |
| Tapetum R | 0.027 | 0.846 | 0.154 | 0.260 | -0.164 | 0.232 | 0.217 | 0.111 | 0.244 | 0.073 | -0.176 | 0.198 |
| Tapetum L | 0.023 | 0.865 | -0.114 | 0.407 | -0.042 | 0.763 | 0.018 | 0.896 | 0.107 | 0.437 | 0.054 | 0.697 |

*****Bolded entries correspond to significant correlations after Bonferroni correction (p<0.00104)*****

**Acronyms: NDI: Neurite Density Index, ODI: Orientation Dispersion Index, FA: Fractional Anisotropy, HDRS: Hamilton Depression Rating Scale, SHAPS: Snaith-Hamilton Pleasure Scale**

**
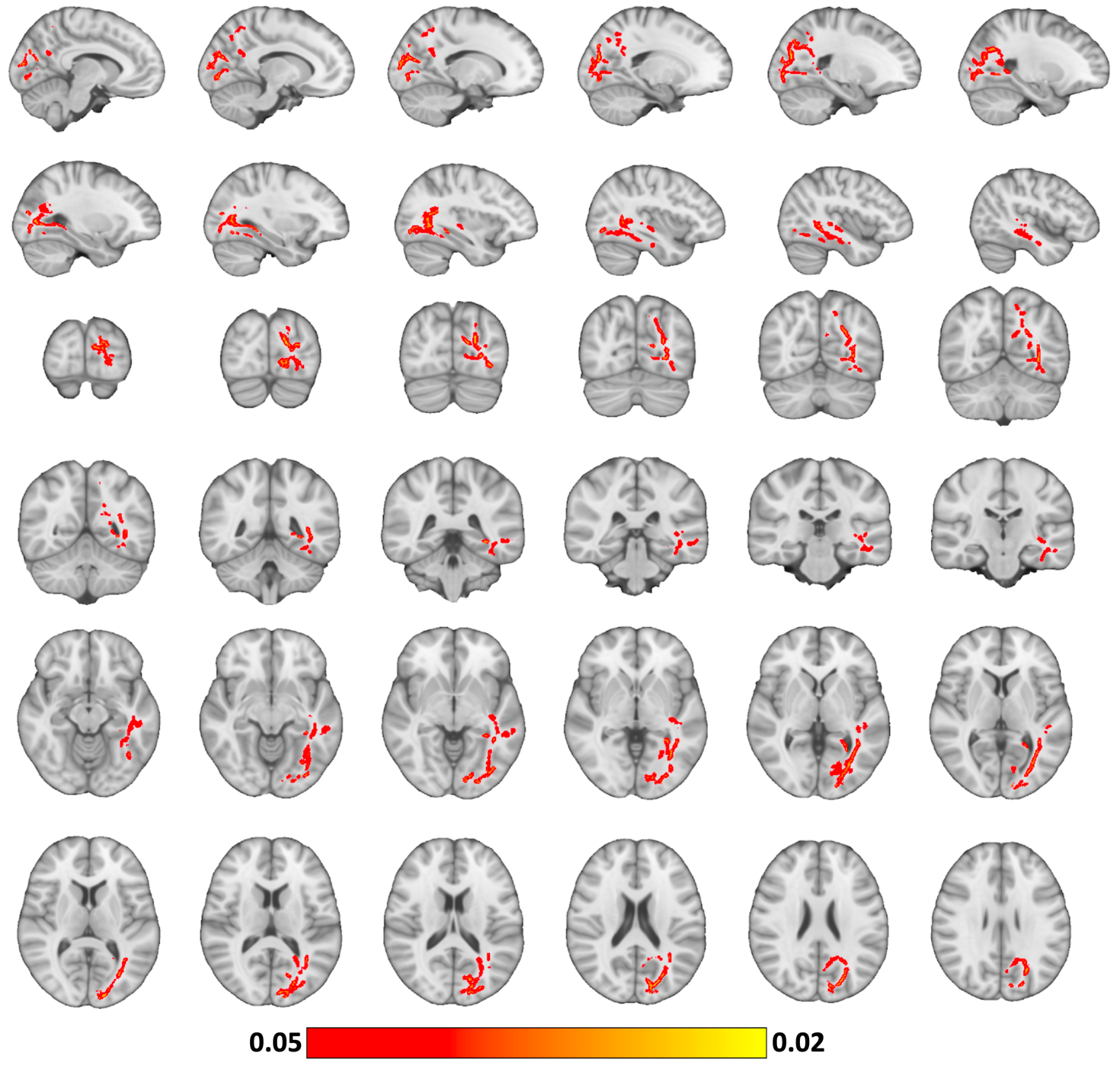
Supplementary Figures:**

**Supplemental Figure 1:** Additional slice views of the significant changes in WM following serial ketamine infusion (SKI), which expands upon the images shown in Figure 3a. Significant voxels are overlaid on the MNI-152 brain, with brighter colors corresponding to greater significance.

**
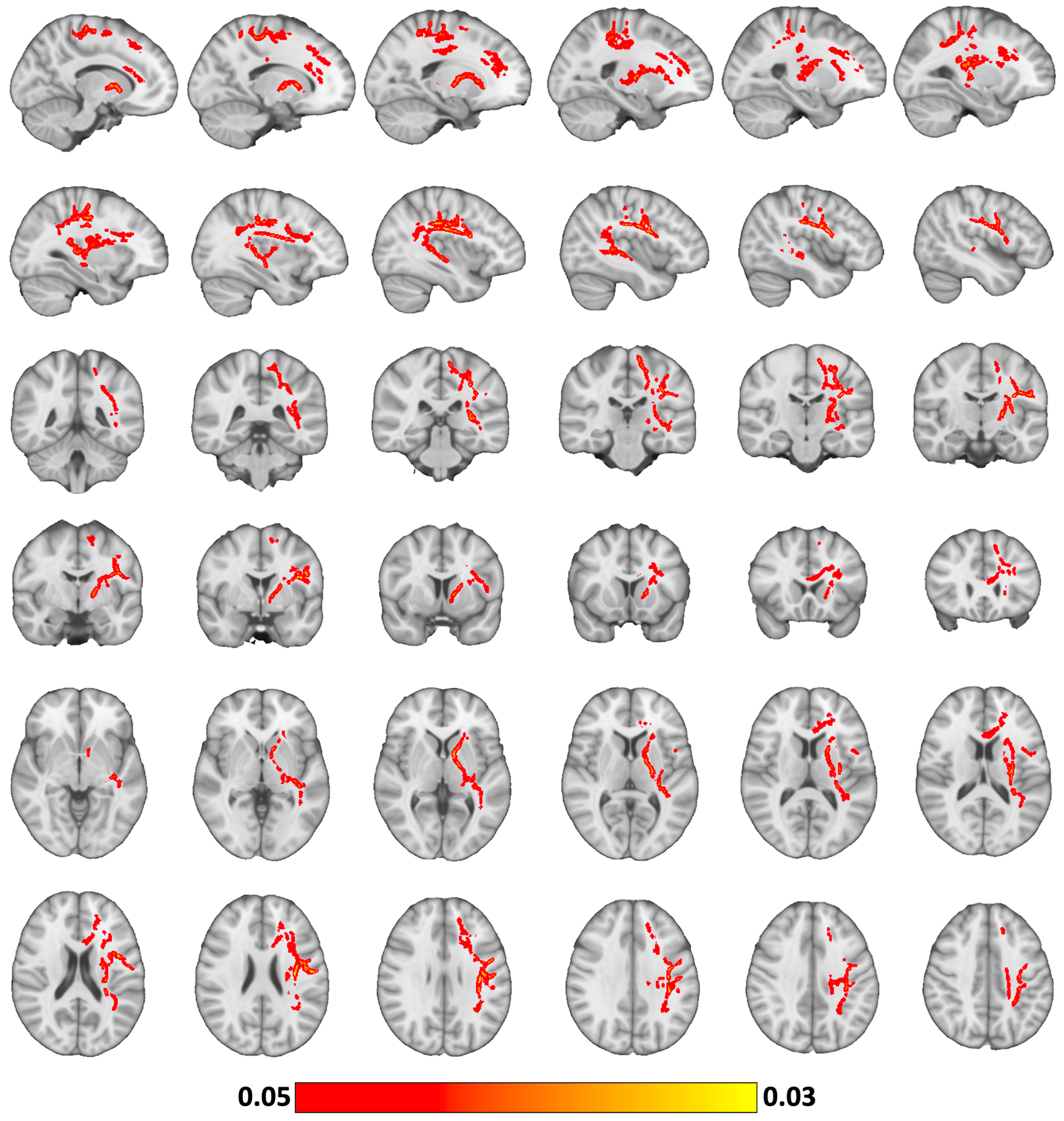
**

**Supplemental Figure 2:** Additional slice views of the regions that showed significant associations with SHAPS following serial ketamine infusion, which expands upon the images shown in Figure 4a. Significant voxels are overlaid on the MNI-152 brain, with brighter colors corresponding to greater significance.


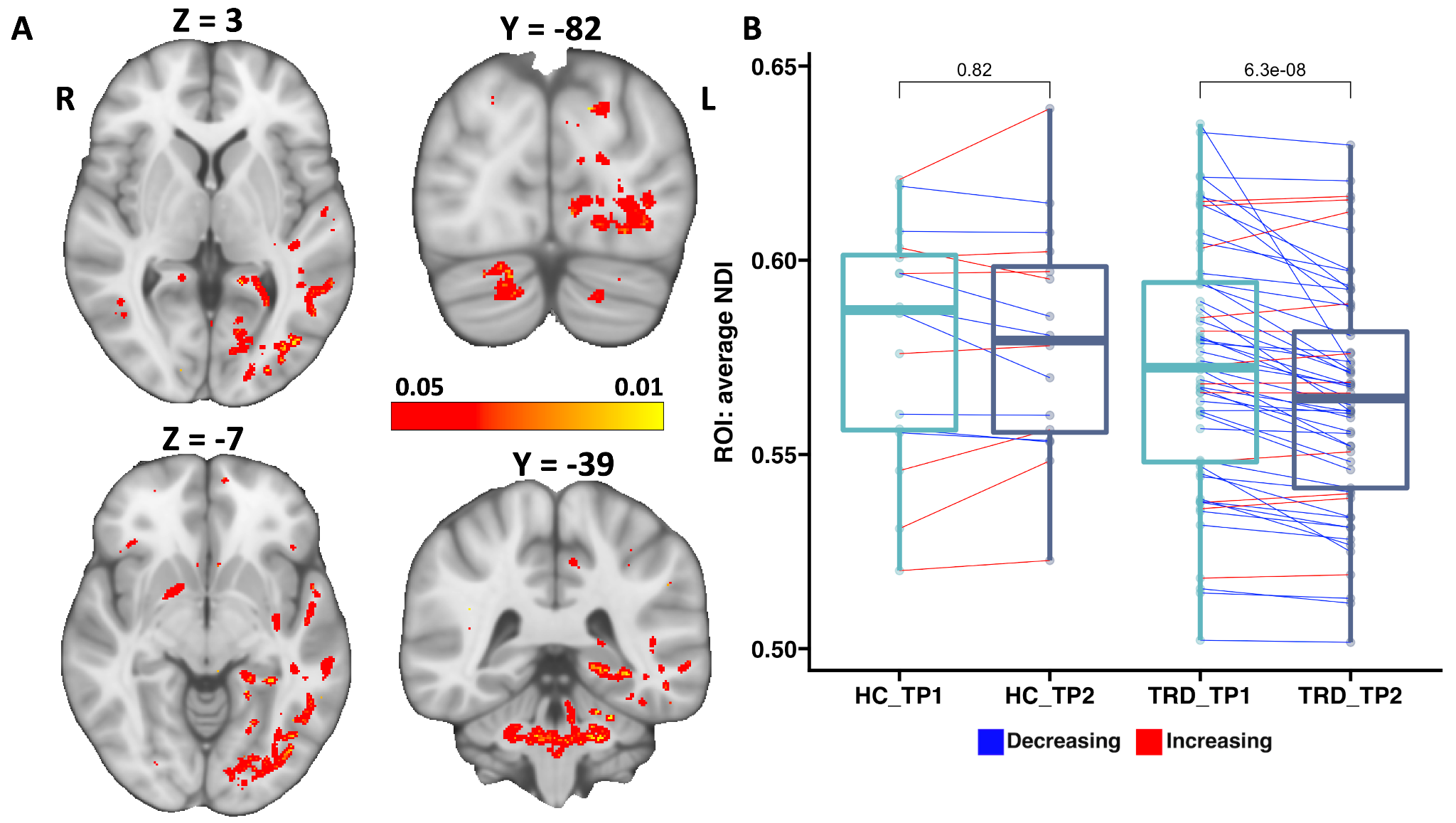


**Supplemental Figure 3:** Comparisons between WM NDI changes in healthy controls over time versus NDI changes in treatment resistant depression following serial ketamine treatment. **(a)** A voxel-level two-sample t-test was performed in *Randomise* to compare NDI change in TRD and HC participants, using age and sex as covariates of no interest. Though voxel-level results of change between HCs and TRD did not survive FWER correction, uncorrected p-value maps with TFCE showed trends in WM regions overlapping with those showing significant decreases following ketamine in TRD (see **Figure 3**). **(b)** Using voxels showing significant effects of ketamine as a statistical ROI, NDI change was computed for each subject and compared between the TRD and HC groups. A significant difference between groups was observed within this statistical ROI (t=-2.954, p=0.0043), with TRD patients showing significantly greater decreases in NDI than HC participants. Boxplots show NDI for TRD and HCs at each timepoint, with lines connecting each subject at both timepoints to indicate the change over time. P-values comparing change across time within each group are shown above each set of box plots.


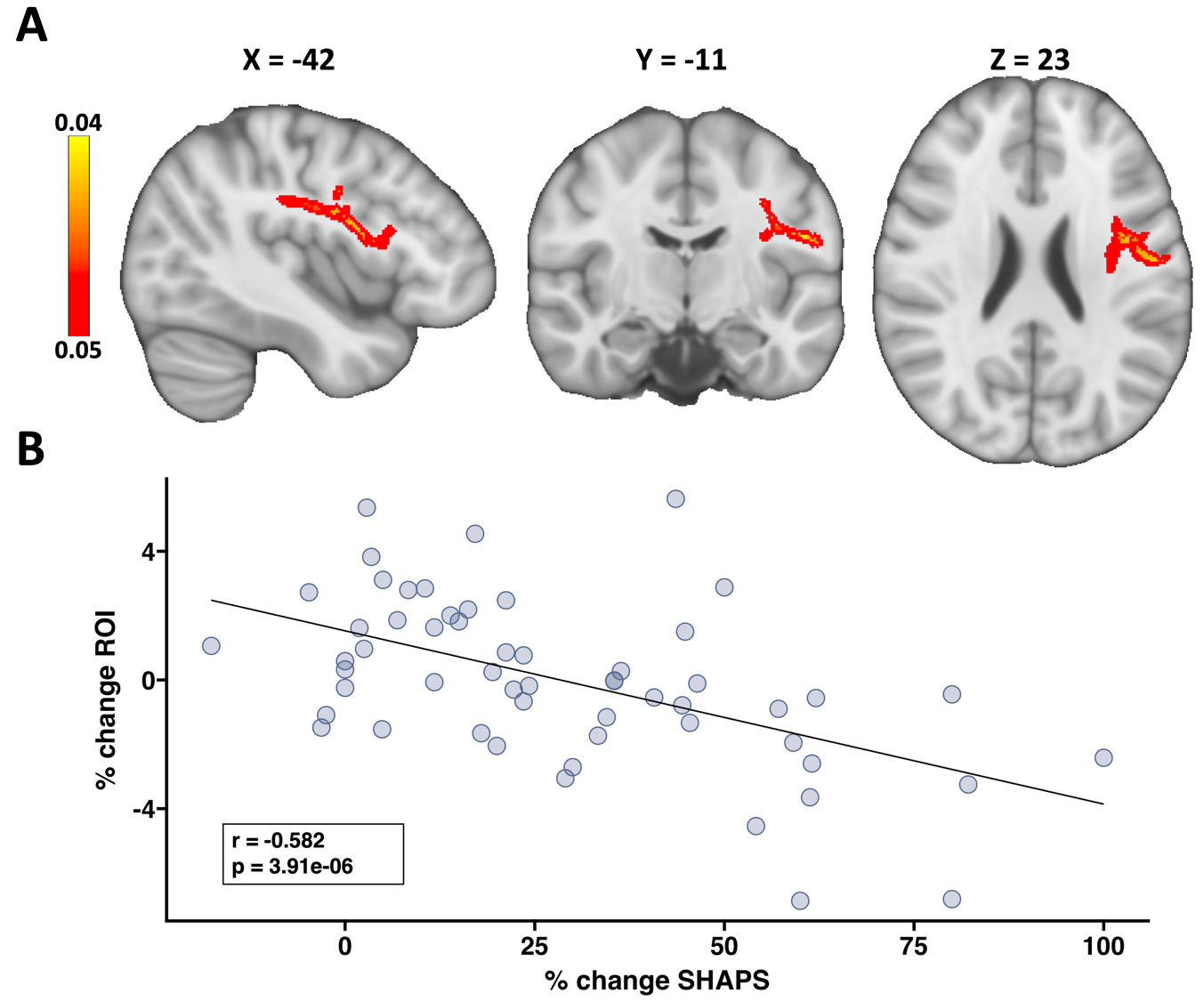


**Supplemental Figure 4:** WM Associations with SHAPS following serial ketamine infusion after removal of outliers. The voxel-wise correlation analysis was repeated after the removal of a single outlier participant. Change in NDI within the left superior longitudinal fasciculus remains significantly negatively correlated with SHAPS, suggesting that greater reductions in NDI within these tracts are associated with greater improvements in anhedonia following ketamine treatment. **(a)** Slice views of MNI-152 T1w brain image with significantly correlated voxels overlaid on top, and coordinates for each slice below. **(b)** The average percent change in NDI within the significant voxels was computed for each subject and plotted against their percent change in SHAPS in order to visualize the associations between change in NDI and anhedonia. R- and p-values are shown above for the correlation between percent change in NDI averaged across significant voxels and percent change in SHAPS.
